# Effects of oral screen exercise on orofacial and pharyngeal activity: An exploratory study using videofluoroscopy and surface electromyography in healthy adults

**DOI:** 10.1002/cre2.538

**Published:** 2022-02-01

**Authors:** Lisa Bengtsson, Hans Dotevall, Lotta Sjögreen, Lena Ragnemalm, Lisa Tuomi

**Affiliations:** ^1^ Region Västra Götaland, Public Dental Service, Mun‐H‐Center Orofacial Resource Centre for Rare Diseases Gothenburg Sweden; ^2^ Department of Otorhinolaryngology, Region Västra Götaland Sahlgrenska University Hospital Gothenburg Sweden; ^3^ Department of Otorhinolaryngology, Head and Neck Surgery, Institute of Clinical Sciences, Sahlgrenska Academy Gothenburg University Gothenburg Sweden; ^4^ Department of Radiology, Region Västra Götaland Sahlgrenska University Hospital Gothenburg Sweden

**Keywords:** deglutition disorders, myofunctional therapy, oropharynx, pharynx, radiography

## Abstract

**Objective:**

The oral screen is a device commonly used for treatment of orofacial disorders. The objective of this exploratory study was to examine the effect of oral screen exercise on the muscle activity in the lips, submental complex, masseter muscle, and kinematic activity of the tongue base, soft palate, pharynx, and larynx in healthy adults. This was compared with the kinematic activity during a dry swallow. It was hypothesized that not only the lip musculature but also other structures in the oral and pharyngeal cavities are activated while using an oral screen device.

**Method:**

Ten healthy subjects used an oral screen during examination with videofluoroscopy and surface electromyography (EMG). Three different instructions for oral screen application and a dry swallow were examined.

**Results:**

The lip muscles showed the highest activity during oral screen exercise. The other muscle groups were activated to a lesser degree. The pattern of activation differed between individuals. Compared with a dry swallow, the range of motion of the tongue base, posterior pharyngeal wall, and the larynx was significantly smaller during oral screen activation. No major differences were found between three different instructions.

**Conclusion:**

This study indicates that the lips and submental complex and, to a lesser degree, oral, pharyngeal, and laryngeal structures are activated with the oral screen, but the pattern of activation varied between individuals. In comparison to the activity during a dry swallow, range of motion during oral screen exercise is small.

## 1 BACKGROUND

Treatment with oral screens has been used in therapy for orofacial disorders since the introduction of the concept “Orofacial Regulation Therapy” by Castillo‐Morales in the 1980s (Lundälv, 1998). According to Castillo Morales, the purpose of the oral screen exercise is to strengthen the buccinator mechanism, a muscle chain involving the lips, cheeks, and pharynx, with more effective lip closure and swallowing as a result (Castillo Morales RC & Haberstock, 1991). Anatomical research by D'Andrea and Barbaix has shown that there is a deep unit composed of musculus buccinator and the inner ring of musculus orbicularis oris, with a superficial unit built up by musculus depressor anguli oris, musculus zygomaticus, musculus risorius, and the outer ring of musculus orbicularis oris (D'Andrea & Barbaix, 2006). The muscles included in these units are closely interrelated (D'Andrea & Barbaix, 2006). The perioral muscles are active in both lip closure and lip rounding. Lip closure and lip control are important for the oral phase of swallowing and saliva management (Lespargot et al., 1993).

The oral screen is an exercise tool made of plastic materials. It consists of a flat screen made of soft or hard plastic, and, usually, the screen is fitted with a handle. The design varies somewhat between different brands and may be custom made according to the anatomy of the individual. The screen is placed inside the lips and pulled against the resistance of the buccinator musculature with the handle. There is no consensus in the literature regarding the actual instruction of use, for example, whether the oral screen exercise should be performed with or without active suction of the lips or with or without active placement of the tongue against the alveolar wall (Faceformer; Hägglund et al., 2019; Sjögreen et al., 2010). Usually, the exercise is recommended to be executed during a few minutes one to three times daily (Hägglund et al., 2019; Sjögreen et al., 2010).

Subsequent research has suggested that oral screen exercises not only improve the strength of the lip musculature (Sjögreen et al., 2010) but also improve oropharyngeal swallowing (Hägg & Anniko, 2010; Hägg & Tibbling, 2015; Hägglund et al., 2019), affect the position of the incisors (Owman‐Moll & Ingervall, 1984; Thüer & Ingervall, 1990) and soft palate closure (Hägg & Tibbling, 2016; Hägg et al., 2015a), reduce gastroesophageal reflux (Hägg et al., 2015b), and improve postural control (Hägg & Tibbling, 2016). Sjögreen et al. noted that the maximal lip force and lip force endurance improved in school aged children with myotonic dystrophia type 1 after oral screen training (Sjögreen et al., 2010). Furthermore, data from studies by Hägg and collaborators indicate that oral screen exercise may have a positive effect on oropharyngeal function and swallowing function in older people and in patients with dysphagia after stroke (Hägg & Anniko, 2008, 2010; Hägg & Tibbling, 2015). However, the precise pattern of activation of different structures and muscle groups in the oral cavity and the pharynx is still unclear. To the best of our knowledge, there are no studies that evaluate the actual movements of the targeted structures.

The aim of this exploratory study was to examine the muscle activity in the lips, submental complex, and masseter muscle, and kinematic activity in the tongue base, soft palate, pharynx, and larynx in healthy adults during oral screen exercise, using videofluoroscopy (VFS) and surface electromyography (sEMG). A secondary aim was to compare the structural movements during oral screen exercise with the activity during a dry swallow using VFS. Three different instructions on how to stabilize the oral screen were compared. It was hypothesized that not only the lip musculature but also other structures in the oral and pharyngeal cavities are activated in a distinctive pattern while using an oral screen device. Moreover, it was hypothesized that the oral screen exercise would induce appreciable activity in oral and pharyngeal structures involved in swallowing.

## 2 MATERIAL AND METHODS

### 2.1 Participants

Ten healthy adults aged 45–59 years were included in the study. The participants were acquainted colleagues with the authors. Five were males and five were females. They all had a stabile occlusion and no increased overjet or open bite. None of the participants reported oral motor, swallowing, or speech problems. No one used neurotropic drugs. All the subjects filled in a form with personal data and information about the above before inclusion.

### 2.2 Material

**Figure 1:**
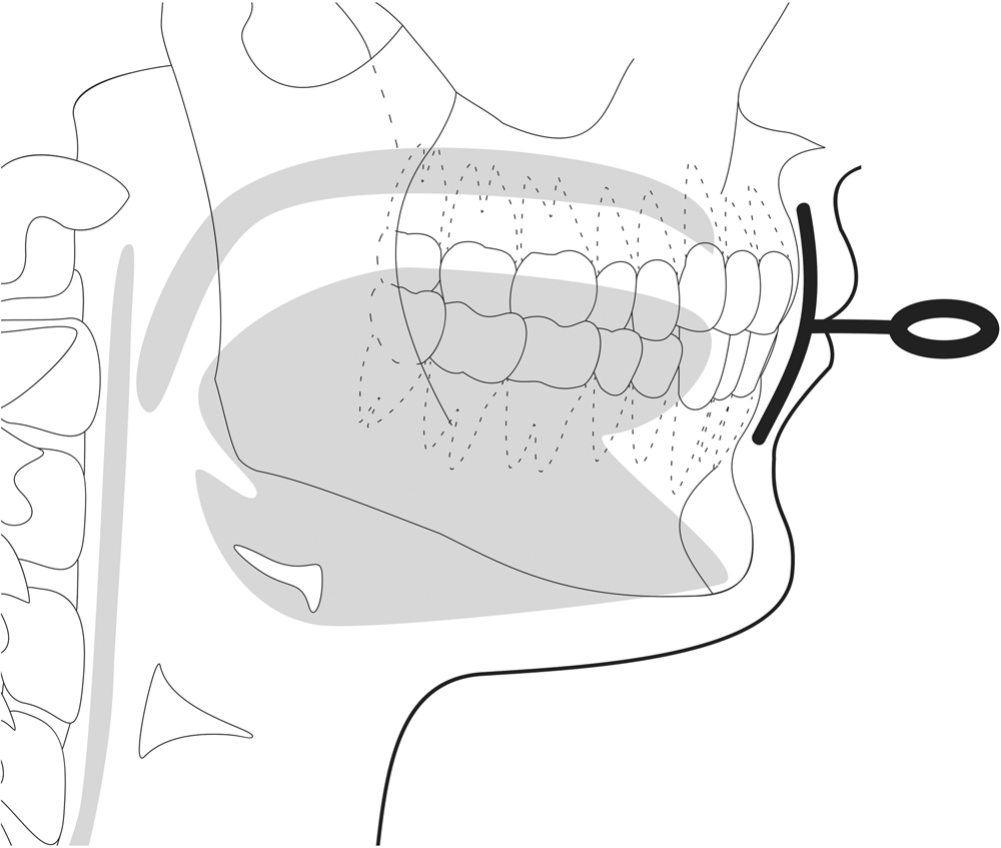
Placement of oral screen between the lips and teeth, with a small handle outside the mouth. Picture by Inga Svensson. The image is used with the permission of the artist

A prefabricated oral screen (Ulmer munskärm®, Forshaga Dentaldepå AB) was used for the study. The Ulmer munskärm® is a semi‐soft oral screen made of polyurethane. The oral screen is placed between the lips and teeth. A small handle on the outside of the screen is used for pulling the device (Figure 1).

### 2.3 Procedure

Before the assessment, maximum lip force was measured in all subjects with the oral screen in place using a power gauge (LF100, Detektor AB). The maximum lip force was obtained when the subject was no longer able to keep the oral screen in place when pulled with increased force traction, measured in Newtons (N). Maximum lip force was measured and documented in a protocol for each participant.

For the measurement during VFS and sEMG, traction of the oral screen with 50% (±10%) of the individual maximum lip force was used. Fifty percent maximum lip force was chosen to enable endurance during the whole examination. The traction was maintained for 5 s and was repeated three times with three different verbal instructions on how to stabilize and retain the oral screen in place during traction for both the VFS and sEMG (i.e., a total of 18 trials). The traction force was monitored with the power gauge to maintain approximately the same traction. If necessary, the participant was given feedback of the traction force. The three instruction was chosen according to previous research and clinical praxis. The instructions were as follows:

1) Hold the oral screen in place with the tongue in a neutral position in the mouth without active suction (“no active suction”).
2) Hold the oral screen in place with the tip of the tongue anchored to the alveolar wall (“tongue anchored to the alveolar wall”).
3) Hold the oral screen in place with active suction (“active suction”).

The three different instructions for activation were performed in a randomized manner in both the VFS and sEMG examinations. All measurements were made at one appointment. The participants were able to rest between the VFS and sEMG assessments in order not to overstrain the musculature.

### 2.4 Videofluoroscopic examination

The VFS examination was performed in the lateral projection, with the digital storage of high‐resolution images (video matrix 1024 × 1024 at a rate of 7.5 frames per second) (Siemens Artis). The field of view included the lips, the tip of the tongue anteriorly, the pharyngeal wall posteriorly, the soft palate superiorly, and the seventh cervical vertebra inferiorly. Before the measurement, a cephalogram in the resting position was captured for all individuals for baseline data. For calibration purposes, a coin of known size was attached under the chin with sticky‐tape.

The VFS examination started with a dry swallow. The image capture during the oral screen exercise began when the subject reached 50% of maximum lip force and continued for about 6 s to include the complete sequence of oral screen traction. This was repeated three times with a short break between the three different instructions, as described above. A speech language pathologist (SLP) gave the instructions, as well as managed the oral screen and power gauge. A gastrointestinal radiologist managed the radiologic equipment and software.

### 2.5 sEMG

A four‐channel sEMG monitor (ME6000 biomonitor; Mega Electronics Ltd) and Ambu BlueSensor NECG surface electrodes (Ambu A/S) were used for the sEMG measurement. The signals were captured and stored for later analysis using MegaWin™ 3.1 MT‐WIN software (Mega Electronics Ltd) on a laptop computer. The sEMG signals were registered from (1) musculus masseter on the left side, (2) the submental complex (i.e., digastric, mylohyoid, geniohyoid, and genioglossus muscles), (3) musculus orbicularis oris superior, and (4) musculus orbicularis oris inferior. Before placing the surface electrodes, the skin was prepared by cleaning with alcohol and, if necessary, shaving. Reference electrodes were placed over bony structures in proximity. The positions of the electrodes were based on palpable and individual anatomical prerequisites. The sEMG recording was initiated just before oral screen exercise and continued for about 6 s for each trial. The sEMG signal during resting state was registered before each separate trial and was used as baseline for the analysis (Fridlund & Cacioppo, 1986).

### 2.6 Analysis of VFS

**Figure 2:**
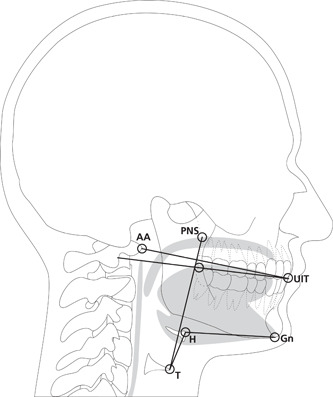
Radiologic landmarks for the VFS analysis: AA, the anterior point of the atlas vertebra; UIT, the edge of the upper incisor teeth; H, anterior edge of the hyoid bone; Gn, gnathion; T, upper anterior corner of the thyroid cartilage; PNS, posterior nasal spina. Picture by Inga Svensson. Used with permission. VFS, videofluoroscopy

Measurements of variables related to movements of the oral and pharyngeal structures were made on representative VFS images at resting state before and steady state during oral screen exercise. Specific radiologic landmarks were chosen to construct references axes perpendicular to or in the direction of the movement of the anatomic structures (Figure 2). The movement amplitude was defined as the difference between the position of the structure at rest compared with the position at steady state during oral screen exercise along the defined trajectory. The following kinematic variables were used for the analysis.

1. Soft palate movement: The distance of movement along an axis from the anterior point of the atlas vertebra (AA) to the lower edge of the maxillary incisor teeth (UIT).
2. Tongue base movement: The distance of movement along an axis from the UIT in line with the lower border of the upper teeth.
3. Posterior pharyngeal wall movement: The distance of movement along an axis from the UIT in line with the lower border of the upper teeth (i.e., the movement at the level of the tongue base).
4. Hyoid movement: The distance of movement of the anterior edge of the hyoid bone (H) relative to the gnathion (Gn; the lowest point of the anterior margin of the mandible).
5. Movement of the larynx: The distance of movement of the upper anterior corner on the thyroid cartilage (T) relative to the posterior nasal spina (PNS; the process formed by the posterior border of the two palatine bones).
6. Thyroidhyoid approximation: The distance of approximation between the upper anterior corner of the thyroid cartilage (T) and the anterior edge of the hyoid bone (H) (Leonard et al., 2000).

The same variables were used for analysis of structural movement during a dry swallow for comparison.

The primary VFS analysis was performed by a gastrointestinal radiologist. Forty percent of the measurements were randomly duplicated for analysis of intra‐rater reliability and 40% of the measurements were analyzed by a second rater for analysis of inter‐rater reliability.

### 2.7 Analysis of sEMG

The sEMG signal was sampled at 1000 Hz and low‐pass filtered at 50 Hz and high‐pass filtered at 450 Hz to remove low‐ and high‐frequency noise. The signal was then smoothed using the root mean square (RMS) method using a time frame width of 0.1 s. As baseline, the mean amplitude of the sEMG signal during 2 s of resting with the oral screen in place was used. The sEMG activity was measured as the mean sEMG amplitude over a time interval of 2 s during the plateau phase of the sEMG signal at steady state during oral screen exercise, beginning from 1 s after the first sEMG peak for each trial.

The sEMG signals were analyzed with MegaWin™ 3.1 MT‐WIN software. The difference between the sEMG signal at baseline and oral screen exercise in millivolts was used as measure of sEMG activity. The mean of the three trials of each three instructions was used for the analysis. Second, a measure of the relative workload of the four muscle groups (musculus orbicularis oris superior, musculus orbicularis oris inferior, the submental complex, and musculus masseter) during oral screen activation was calculated. The workload measurement was defined as the area under the curve of each EMG channel over the same time interval of 2 s during the sEMG plateau phase during oral screen exercise. The percentage of the total workload for each muscle group was calculated.

### 2.8 Statistical analysis

Analyses were performed using the Statistical Package for the Social Sciences (SPSS, IBM, Sweden) version 25 for Mac and Microsoft Excel for Mac, version 14.00. Due to the non‐normal distribution of the data, nonparametric tests were used. All the tests were two‐tailed with the level of significance set at *p* < .05. The median, min, and max values were extracted for descriptive purposes. To explore differences between two groups, for example, possible differences between men and women, the Mann–Whitney *U* test was used. For comparisons between the three different instructions, Friedman's ANOVA with post hoc pairwise comparisons with Bonferroni correction of *p* values, was used. The data are reported in descriptive measurements (e.g., range of motion) and as a qualitative analysis with a comparison of the three different instructions and a dry swallow. Inter‐ and intra‐rater reliability were calculated using intra‐class correlation (ICC), a two‐way mixed model and absolute agreement. ICC results are presented as either “single” or “average” measurements. Based on the 95% confidence interval of the ICC estimate, values less than 0.5, between 0.5 and 0.75, between 0.75 and 0.9 and greater than 0.90 are indicative of poor, moderate, good and excellent reliability, respectively (Koo & Li, 2016).

### 2.9 Ethical considerations

The study was conducted in accordance with the Declaration of Helsinki and was approved by the Regional Ethical Review Board in Gothenburg, Sweden. The study has received approval from The Swedish Radiation Safety Authority. All the participants gave their informed consent before inclusion in the study.

## 3 RESULTS

### 3.1 Maximum lip force

The maximum lip force differed significantly between the three different instructions (Table 3). The highest force was obtained during active suction of the oral screen (instruction #3) in all participants except one. One individual displayed the highest lif force during instruction #1 (no active suction). There were no statistically significant differences between men and women regarding maximum lip force (Table 1).

**Table 1:** Maximum lip force in the group of healthy adults

Lip force (N) (median and range)			*p* values			
Instruction # 1 (no suction)	Instruction # 2 (anchoring the tip of the tongue)	Instruction # 3 (active suction)	Instruction #1 versus #2 versus #3	Instruction # 1 versus #2	Instruction # 2 versus #3	Instruction # 1 versus #3
17.5 (5–28)	11 (4–40)	29 (15–51)	.001	ns	.001	.042

*Note*: Comparison between three different instructions on how to stabilize the oral screen.

Abbreviation: ns, nonsignificant (*p* > .05).

### 3.2 VFS

**Figure 3:**
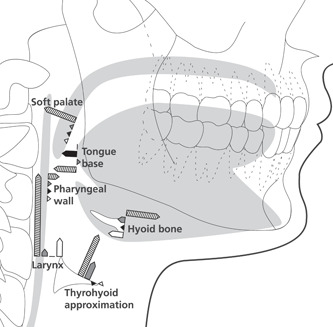
Median range of motion of the soft palate, posterior pharyngeal wall, tongue base, hyoid bone, larynx and thyrohyoid approximation during dry swallowing (hatched) and oral screen activation on the three different instructions, no active suction (instruction 1: white), anchoring the tip of the tongue against the alveolar wall (instruction 2: black) and active suction (instruction 3: gray)

For all participants, motion was observed in all the examined structures during the oral screen exercise on all instructions, albeit the range of motion was small (Table 2) (Figure 3). The pattern of movement varied between participants. In some cases, participants moved the examined structures in different directions. For example, two participants lowered their hyoid bone, while the others raised their hyoid bone during the oral screen exercise. Differences between individuals regarding strategies to stabilize the oral screen against resistance applies to all anatomical structures and in all three instructions. There were no statistically significant differences in range of motion between the three different instructions.

**Table 2:** Range of motion during oral screen exercise

	Range of motion (mm)				*p* values			
Anatomic structure	Instruction # 1 (no suction)	Instruction # 2 (anchoring the tip of the tongue)	Instruction # 3 (active suction)	Dry swallow median (min‐max)	Instruction #1 versus #2 versus #3	Dry swallow versus instruction # 1	Dry swallow versus instruction # 2	Dry swallow versus instruction # 3
Soft palate	1^a^ (−1−8)	1^a^ (−6−6)	1^a^ (−6−6)	9 (4−17)	ns	ns	.015	.044
Pharyngeal wall	−1^a^ (−4−1)	−1^a^ (−5−3)	−1^a^ (−3−5)	−3 (−6 to −1)	ns	.001	.015	ns
Tongue base	0 (−6 −6)	−4^a^ (−14−6)	1 (−9−7)	−6 (−16 to −3)	ns	ns	<.001	ns
Hyoid	4 (−2−7)	1 (−4−8)	2 (−3−13)	−9 (−14 to −4)	ns	ns	.001	.026
Larynx	−5^a^ (−12−30)	0 (−6−23)	−2^a^ (−7−25)	−22 (−36 to −19)	ns	.011	ns	.011
Thyrohyoid approximation	−1^a^ (−13−10)	1 (−2−7)	−5^a^ (−12 to −6)	−12 (−16 to −10)	ns	.002	ns	<.001

*Note*: Comparison between three different instructions on how to stabilize the oral screen and a dry swallow. Range of motion in millimeters (median and range). A positive value in soft palate movements indicates elevation of the soft palate. A negative value for the pharyngeal wall indicates anterior movement. A negative value for the tongue base indicates anterior‐superior movement. A negative value for hyoid movement indicates anterior‐superior movement. A negative value for laryngeal movements indicates laryngeal elevation. A negative value for the thyroid‐hyoid indicates approximation of the thyroid‐hyoid.

Abbreviation: ns, nonsignificant.

^a^ The value on oral screen exercise indicates movements in the same direction as movements in swallowing.

The range of motion of the oral and pharyngeal structures and the larynx was significantly larger during the dry swallow in comparison to the oral screen exercise in most cases (Table 2 and Figure 3). The largest difference was seen for laryngeal movement. During the dry swallow the median laryngeal elevation for the dry swallow was 22 mm, while during the oral screen exercise the median range of motion of the larynx varied between a downward movement of 5 mm and no movement (0 mm).

### 3.3 Results of sEMG

The submental complex was significantly more activated during instruction 3 (active suction) compared with instruction #2 (anchoring the tip of the tongue against the alveolar wall) (*p* = .022) (Table 3). Other than that, there were no statistically significant differences between the various instructions. The greatest activation was observed in the lower lip (musculus orbicularis oris inferior), followed by the activation of the upper lip (musculus orbicularis oris superior) on all instructions. For all participants, activation was observed in all the examined muscles on all instructions.

**Table 3:** Median and range values for muscle activation measured with sEMG in millivolts (mV) during oral screen exercise on three instructions and pairwise comparisons between instructions in 10 healthy adults

	sEMG activity (mV)			*p* values			
	Instruction # 1 (no suction)	Instruction # 2 (anchoring the tip of the tongue)	Instruction # 3 (active suction)	Instruction #1 versus #2 versus #3	Instruction #1 versus #2	Instruction #2 versus #3	Instruction #1 versus #3
Musculus masseter	36 (12−187)	43 (8–121)	50 (11−110)	.273	–	–	–
Submental complex	94 (30−188)	88 (46–163)	119 (40–213)	.027	ns	.022	ns
Musculus orbicularis oris superior	179 (87–398)	173 (104–337)	184 (108–372)	.082	–	–	–
Musculus orbicularis oris inferior	283 (110−511)	208 (72−430)	248 (75−473)	.061	–	–	–

Abbreviations: –, not applicable; ns, nonsignificant (*p* > .05); sEMG, surface electromyography.

**Figure 4:**
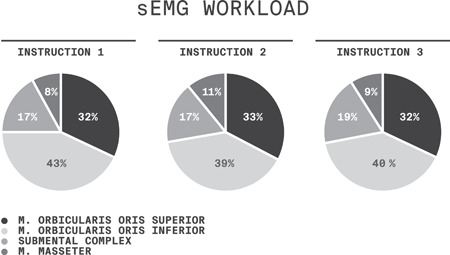
Mean relative sEMG workload in percent. Instruction 1, no active suction; Instruction 2, anchoring the tongue against the alveolar wall and; Instruction 3, active suction. sEMG, surface electromyography

The workload analysis of the four muscles or muscle groups showed a similar distribution on all three instructions (no active suction, anchoring the tongue against the alveolar wall, and active suction) (Figure 4). The orbicularis oris muscle, superior and inferior, contributed 72%–75% of the total workload, the submental complex 17%–19%, and the masseter 8%–11%.

### 3.4 Reliability

Inter‐ and intra‐rater reliability were calculated for the videofluoroscopic examinations by two experienced judges using ICC, a two‐way mixed model. The intra‐rater reliability revealed an ICC (single measurement) of 0.92 (95% confidence interval [CI]: 0.89–0.94), while inter‐rater reliability (single measurement) was 0.89 (95% CI: 0.83‐0.94).

## 4 DISCUSSION

The aim of this study was to explore the activity in the oral cavity and pharynx during oral screen exercise, using VFS and sEMG in healthy adults. The data indicate that the lip musculature and submental complex and, to a lesser degree, oral, pharyngeal, and laryngeal structures are activated with the oral screen, but the pattern of activation varied between individuals. The VFS examination showed that the participants used different techniques for holding the oral screen against resistance, while the sEMG revealed that the lips, in particular the lower lip, was most active. Anatomical structures involved in swallowing appear to be active during the oral screen exercise in most subjects, but in comparison to the movements during a dry swallow the range of motion was significantly smaller and in some cases in opposite direction compared with the swallow.

It is not possible to compare the data of maximum lip force in this study to the results in other studies (Wertsén & Stenberg, 2017a, 2017b), as the design of the oral screen and the way lip force is measured have an impact on the results. According to Wertzén and Stenberg (2017a, 2017b), lip force increases in relation to the projected area of the screen but vary between individuals. The same problem with comparisons of results applies to lip force on different instructions. Sucking generally increases measured lip force (Wertsén & Stenberg, 2017b), which was confirmed in this study. The results for lip force showed no differences regarding gender, which is in line with previous research (Wertsén & Stenberg, 2017a). However, the study group of this exploratory study is too small to draw any conclusion regarding possible gender differences.

Similarly, the design of the oral screen may have an impact on the oral and pharyngeal activity. The choice of oral screen in this study was justified by the fact that the Ulmer munskärm® is commonly used by clinicians in Sweden and is one of the least expensive, prefabricated oral screens on the Swedish market. Further studies on the effect of oral screens with different shapes and sizes on the activity in the mouth and pharynx are warranted. A larger oral screen or another design might activate the muscles more and produce a greater range of motion. In this study there were no obvious differences between the three instructions on a group level regarding the movement of oral and pharyngeal structures or the sEMG activity.

Previous published data indicate that oral screen exercise may, among other things, improve swallowing ability in older people and after stroke (Hägg & Anniko, 2008, 2010; Hägg & Tibbling, 2015; Hägglund et al., 2019). The hypothesis that the oral screen exercise would induce appreciable activity in structures involved in swallowing could not be verified in this study. During pharyngeal swallowing the tongue base is moved posteriorly, the soft palate elevates, and the hyoid bone and larynx moves upwards and anteriorly (Logemann 1998; Shaker et al., 2016). Even though the oral screen appeared to activate muscles in the mouth and pharynx somewhat, the range of movement in all the examined structures was significantly smaller than during a dry swallow. Moreover, the direction of movement during use of the oral screen was in opposite direction compared with swallowing in some individuals, for example, lowering of the larynx and hyoid bone. Therefore, the results of this study make it difficult to draw any definite conclusions regarding the possible effect on swallowing ability using oral screen exercise.

Reduced lip closure affects swallowing (Lespargot et al., 1993). The results from the sEMG show that the lip muscles are activated during oral screen exercise. Of the four muscle groups compared in workload analysis, the lip muscles accounted for the greatest activity. Research has shown that oral screen exercise is able to improve lip force (Lundälv, 1998). This may perhaps be one explanation to the results of other studies regarding the effect of the oral screen exercise on swallowing (Hägg & Anniko, 2008, 2010; Hägg & Tibbling, 2015; Hägglund et al., 2019) Further research is required to determine whether this is the case.

During oral screen exercise, it is likely that both supra‐ and infra hyoidal muscles work together to stabilize the hyoid bone and thus the structures of the oral cavity, pharynx, and larynx. As the supra‐ and infrahyoidal muscles contract, activation does not necessarily lead to elevation or depression of the hyoid bone. It is possible that all participants activated the muscles for the joint strategy to stabilize the hyoid bone. The fact that some subjects had an upward movement in the larynx and others downward, may be due to the fact that there was not always a total balance in strength between the muscles involved. Proper stabilization of the hyoid bone is probably important for safe swallowing. Training of the stabilization of the hyoid bone could then be an important component of swallowing rehabilitation.

Posterior movement of the hyoid bone was noted during oral screen exercise on all three instructions. One possible explanation is that the suprahyoid muscles are more active than the submental muscle complex during oral screen exercise. This indicates that the activation pattern during oral screen exercise is complex and differs from the muscle activation during swallowing. This result requires further investigation.

Statistically significant difference was found between range of motion during oral screen exercise and range of motion on dry swallow in the majority of the measurements. Larger movements were found on dry swallow than any of the measured movements in VFS. According to Kraaijenga et al. (2015), superior hyoid range of motion varies between 10 and 15 mm for healthy elderly subjects swallowing thin liquid. The range of motion of the hyoid bone on dry swallow in this study is smaller, median 9 mm. The results from this study thus indicate that oral screen exercise does not induce the same pattern of movement as during swallowing.

Muscle overload, among other parameters, is said to be necessary to cause physiological changes and is crucial for the successful rehabilitation of other muscle groups, such as the limbs and suprahyoid upper esophageal sphincter (Gandevia, 2001). A sign of muscle overload is neuromuscular fatigue induced by the exercise (Burkhead et al., 2007). The results from VFS do not appear to provide support for the hypothesis that oral screen exercise imposes a muscle overload on the structures that are important for swallowing, even though several participants mentioned that their lips became tired between the measurements. This study did not investigate muscle overload, and any conclusion based on these reports can therefore not be drawn.

Oral screen exercise can be regarded as a form of static, nonswallowing exercise. Whether there is a transfer from increased strength in static training to improved function has been discussed, as well as nonswallowing exercises for improved swallowing. According to previous literature, there are several parameters that should be included in training to achieve improved function. They are specificity (to become better at a particular skill, you must perform that skill), overload and intensity (a larger than normal load is required for training adaptation), progression (a gradual and systematic increase in the workload over a period of time), initial values (those with the lowest level of skills have the greatest scope for improvement), reversibility (“use it or lose it”) and transfer and diminishing returns (decreasing the expected degree of improvement as individuals become fit) (Ammann et al., 2014; Burkhead et al., 2007; Langmore & Pisegna, 2015). Based on the above, we should once again highlight the question of how oral screen exercise, as a nonswallowing exercise, may improve swallowing. Moreover, as pointed out by Langmore and Pisegna (2015), how is “a better swallow” defined? Is intervention using dry swallowing a better exercise than oral screen exercise, as it is more specific? If the goal of oral screen exercise is increased lip closure and improved lip strength, as this makes it easier to swallow, parts of the oral screen exercise can be regarded as specific and the training can be organized according to the above principles. Furthermore, people who are unable to eat orally might be able to perform nonswallowing exercises. In these cases, nonswallowing exercises, such as oral screen training, could be an important intervention. However, it is important, in both intervention and research, to ask the question: what do we want to improve and why?

In this study, the participants were healthy adults. The way the oral screen affects a person with orofacial dysfunction requires further research. VFS was used to measure range of motion during oral screen exercise. Other possible techniques for examining this, such as ultrasound and fiber endoscopy, were considered. VFS was chosen as it is a validated method for the objective assessment of all phases of swallowing physiology (Logemann, 1998).

### 4.1 Limitations

The number of participants is a potential limitation in this study, but the number was considered sufficient as a basis for further studies on the physiology of oral screen exercise. A larger group of participants may have produced more solid results. In this study 50% of maximum lip force was chosen to enable the participants to maintain the traction throughout the examinations. However, different opinions exist regarding how much traction should be used in oral screen exercise. It is possible that a higher proportion of maximum lip force would have yielded different results. For future studies it might be of interest to compare range of motion and muscle activation using different levels of traction. Another possible limitation, mainly considering the assessment of the dry swallow, is the use of 7.5 frames per second for the videofluorscopic evaluation. The frame rate was chosen since the principal focus was on the activity during steady state during the use of the oral screen. A higher frame rate is generally considered the standard when evaluation swallowing function.

The use of sEMG is limited in that it is difficult to distinguish the activity in one muscle from another (Palmer et al., 1999). The muscles examined in this study are closely interrelated, according to research by D'Andrea and Barbaix (2006). This is the reason why one channel was chosen for the submental complex, rather than examining isolated muscles with needle EMG. The same procedure was used earlier by Palmer et al., among others (Palmer et al., 1999).

### 4.2 Clinical implications

This study has increased the knowledge regarding the physiologic mechanisms and possible effect of oral screen exercising in a clinical context.

## 5 CONCLUSIONS

Using an oral screen activates muscles and cause structure movements in the oral and pharyngeal cavities, as was shown in both the VFS and the sEMG examinations. However, the impact of oral screen exercise in swallowing seems low, since only minimal motion of swallowing‐related structures are demonstrated in the study. Compared with the movements seen in a dry swallow, the range of motion during the oral screen exercise is small. The participants exhibit different techniques and patterns to stabilize orofacial and pharyngeal muscles in order to hold the oral screen against resistance. The different instructions for oral screen exercise appear to play a minor role regarding the range of motion and muscle activity.

## CONFLICT OF INTERESTS

The authors declare that there are no conflict of interests.

## AUTHOR CONTRIBUTIONS

*Conceptualization*: Lisa Bengtsson, Hans Dotevall, Lotta Sjögreen and Lisa Tuomi. *Study design, data acquisition and manuscript preparation*: All authors. *Data analysis*: Lisa Bengtsson, Lisa Tuomi, Hans Dotevall, Lena Ragnemalm. *Interpretation*: Lisa Bengtsson, Hans Dotevall, Lisa Tuomi, Lotta Sjögreen. All authors read and approved the final manuscript.

## Data Availability

Data not available due to privacy/ethical restrictions.
